# Molecular and morphological evidence supports transferring *Sacosperma* (Rubiaceae, Rubioideae) from Spermacoceae to Knoxieae

**DOI:** 10.3897/phytokeys.272.179527

**Published:** 2026-03-26

**Authors:** Brecht Verstraete, Olivier Lachenaud, Petra De Block, Arne Mertens, Steven B. Janssens, Judith R. Schepers, Steven Jansen, Elmar Robbrecht

**Affiliations:** 1 Meise Botanic Garden, Meise, Belgium Meise Botanic Garden Meise Belgium; 2 Leuven Plant Institute, Department of Biology, KU Leuven, Leuven, Belgium Institute of Botany, Ulm University Ulm Germany; 3 Institute of Botany, Ulm University, Ulm, Germany Leuven Plant Institute, Department of Biology, KU Leuven Leuven Belgium

**Keywords:** Africa, biogeography, climate niche, palynology, *
Rhodopentas
*, seed morphology, taxonomy, wood anatomy

## Abstract

The genus *Sacosperma* (Rubiaceae) comprises two species of lianas occurring in tropical African rainforests. It is currently placed in tribe Spermacoceae, but morphological evidence suggests that placement in tribe Knoxieae might be more appropriate. This study presents the first molecular analysis of *Sacosperma*, which supports its placement within Knoxieae, where it is sister to the East African genus *Rhodopentas*. A close relationship between these two genera has never been considered before, and molecular data alone cannot resolve whether they should be treated as distinct genera. To address this, we provide a comparative analysis of *Sacosperma* and *Rhodopentas* based on morphology, anatomy, biogeography, and climate preference. The recognition of *Sacosperma* and *Rhodopentas* as two separate genera is justified, given a number of morphological differences (e.g., habit, imperforate tracheary elements, domatia, stipules, inflorescences, flower size and color), their biogeography (West and Central African vs. East African), and climate preferences (broad vs. narrow climate envelope). *Sacosperma* and *Rhodopentas* share a common ancestor that has likely diverged morphologically in response to differing ecological conditions.

## Introduction

The tropical African genus *Sacosperma* G.Taylor (Rubiaceae) includes two species of climbers from West and Central African rainforests. While the most recent classification of the family (Razafimandimbison and Rydin 2024) places the genus in tribe Spermacoceae within subfamily Rubioideae, some of its morphological characters suggest an affinity with tribe Knoxieae of the same subfamily. In particular, *Sacosperma* has 5-merous flowers, which are paired and helicoidally arranged along the inflorescence axes and often have unequal calyx lobes, characters that are very rare in Spermacoceae (Groeninckx et al. 2009) but common in Knoxieae. This led to the genus being provisionally included in the list of Knoxieae genera in the introduction to the family Rubiaceae in the "Flore d’Afrique centrale" (Robbrecht et al. 2020), though without further comments. The genus has to date never been included in a molecular phylogenetic analysis, and its placement therefore remains enigmatic.

In this paper, we investigate whether the hypothesis that *Sacosperma* belongs to Knoxieae is well founded. To do so, we present the first sequence data for *Sacosperma*, proving that the genus is sister to *Rhodopentas* Kårehed and B.Bremer within Knoxieae. These two genera have never been considered closely related. We conduct a comparative analysis of the morpho-anatomy, pollen morphology, and wood anatomy of the two sister genera and examine how the characters of *Sacosperma* align with those of Knoxieae and, to some extent, Spermacoceae, the tribe where it was placed until now. In addition, we investigate whether these genera differ in terms of geographic distribution and climatic preference.

## Taxonomic history

A few preliminary remarks on tribal concepts in Rubiaceae are warranted before going into detail about the taxonomy of the genus *Sacosperma*. Traditionally, most herbaceous genera of subfamily Rubioideae were placed in three large tribes: Knoxieae and Spermacoceae, each characterized by a solitary ovule in each ovary locule, pendulous or erect, respectively, and Hedyotideae, having many ovules in the locules (e.g., Robbrecht 1988). Molecular studies (Andersson and Rova 1999; Bremer and Manen 2000; Bremer 2009) later showed that Knoxieae and Spermacoceae required a broader circumscription, each incorporating part of the genera previously included in Hedyotideae. We use “Hedyotideae” (in quotation marks) when reference to the former tribal concept with multiovulate taxa is necessary.

### Sacosperma

*Sacosperma* was originally described under the name *Peltospermum* Benth., with a single species, *P. paniculatum* Benth. (Bentham 1849: 400). It was initially placed in the tribe Rondeletieae, a concept that was, at the time, highly artificial. Bentham (1849: 401) recognized an “evident affinity” between *Peltospermum* and *Lerchea* L., suggesting that both genera, along with several others, required re-examination of their tribal placement in Rondeletieae. Subsequent work has indeed led to the exclusion of both genera from Rondeletieae, although *Lerchea*, now placed in Ophiorrhizeae, is not closely related to *Sacosperma* (Razafimandimbison and Rydin 2024).

*Peltospermum* was subsequently treated as a synonym of *Hedyotis* sect. *Diplophragma* Wight & Arn. (Hooker 1873: 57) and then of *Oldenlandia* L. (Hiern 1877: 53; Schumann 1891: 26; Hutchinson and Dalziel 1931: 132), where its only species was renamed as *Oldenlandia peltospermum* Hiern. Both *Hedyotis* L. and *Oldenlandia* were then included in “Hedyotideae.”

Taylor (1944) resurrected the genus and noted that *Peltospermum* Benth. is an illegitimate name, preceded by *Peltospermum* DC. (1838) and *Peltospermum* Moq. (1840). To resolve this, he provided the substitute name *Sacosperma* and the new combination *Sacosperma paniculatum* (Benth.) G.Taylor. He also recognized that *Pentas parviflora* Benth. shared several characters with *S. paniculatum*, “in habit, structure of inflorescence, and form of flowers,” and added a second species to the genus, *Sacosperma parviflorum* (Benth.) G.Taylor. He argued that the climbing woody habit of the genus made its placement in *Oldenlandia* untenable, despite both genera sharing septicidal capsules. He observed that the inflorescences of *Sacosperma* resemble certain species of *Bertiera* Aubl., but emphasized its “considerable resemblance to *Pentas* Benth., particularly in the tendency to have unequal calyx lobes.” Since *Pentas* is now placed in Knoxieae, Taylor (1944) was the first to suggest a link between *Sacosperma* and that tribe. He was also the first to describe the distinctive fenestrated corolla, noting the “more or less prominent papillose grooves on the lines of fusion of the petals.”

Bremekamp (1952) dealt with *Sacosperma* in his monograph of African “Oldenlandieae,” since its type species was at one time considered to belong to *Oldenlandia* (see above). He extensively commented on the historical placement of the genus in Rondeletieae, arguing that the presence of raphides and the absence of pits in the inner tangential wall of the exotestal cells were important arguments for excluding it from that tribe. Bremekamp considered *Sacosperma* to be a distinct genus, noting its isolated position within “Hedyotideae” due to the free stipules and the peculiar structure of the inflorescence. In his taxonomic treatment, he added a third taxon to the genus, *Sacosperma paniculatum* var. *pubescens* Bremek. Verdcourt (1958) likewise placed *Sacosperma* in “Hedyotideae.”

**Figure 1.**
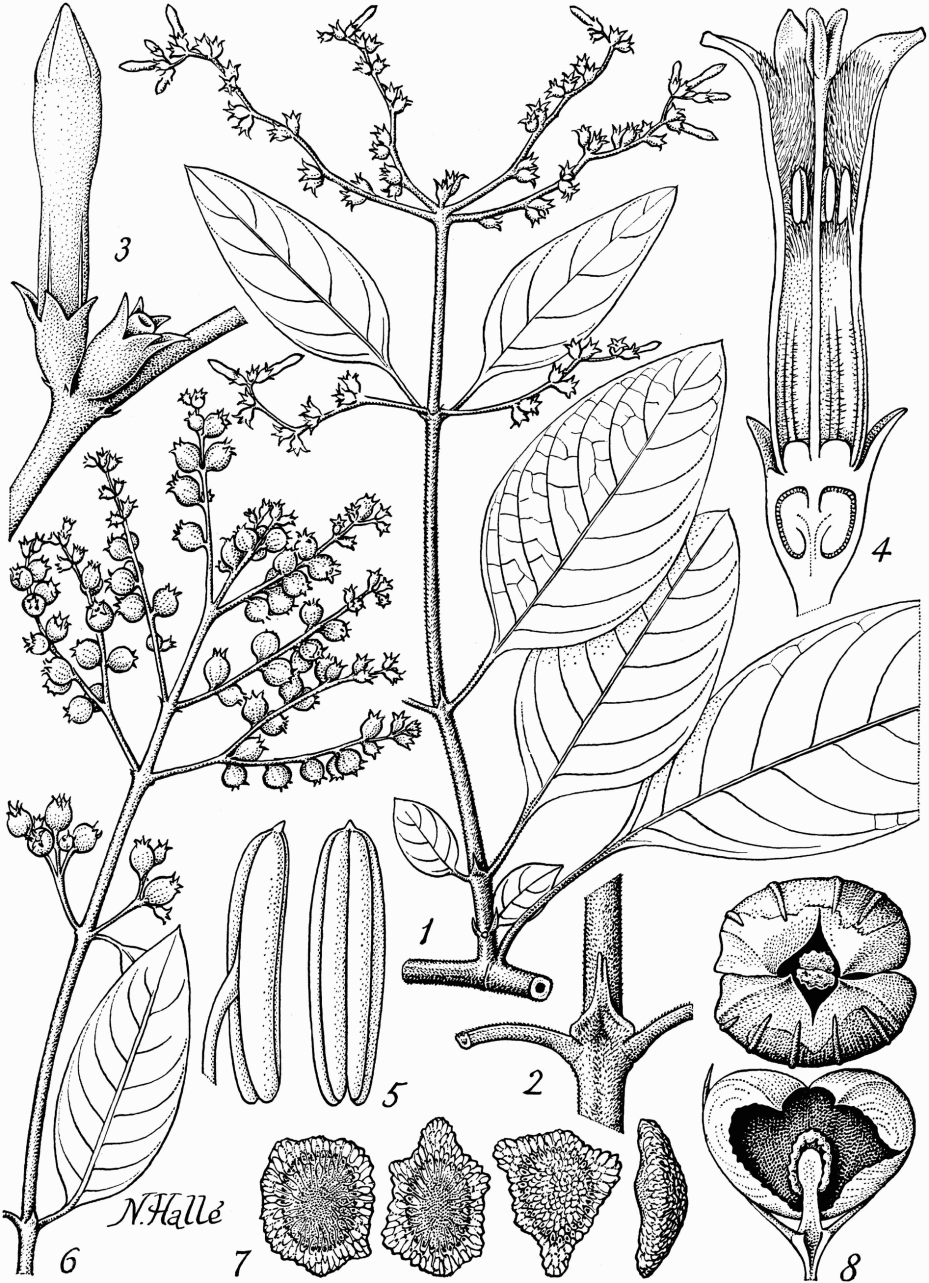
Illustration of *Sacosperma paniculatum*. **1**. Lateral flowering twig showing anisophylly. **2**. Node with bidentate stipule. **3**. Pair of flowers. **4**. Longistylous flower in longitudinal section. **5**. Two views of a stamen. **6**. Infructescence. **7**. Seeds. **8**. Old fruit seen from above and inside view of one valve. Drawing by Nicolas Hallé, reproduced from Hallé (1966), with permission.

Hallé (1961) expressed doubt about the placement of *Sacosperma*. He discussed the genus within the context of his very broadly defined tribe Mussaendeae, which also included *Pauridiantha* Hook.f. and tribe Sabiceeae. Ultimately, he concluded that *Sacosperma* occupied an isolated position between “Hedyotideae” and Mussaendeae. His arguments for an affinity with Mussaendeae were vague, stating only that the genus “présente de nombreux caractères qui l’apparentent à la tribu des Mussaendeae” (“has many characters that relate it to the Mussaendeae tribe”). However, he was more explicit regarding similarities with “Hedyotideae,” particularly *Otomeria* Benth.: “la structure de l’inflorescence et l’aspect du calice l’apparentent nettement au genre *Otomeria*” (“the structure of the inflorescence and the aspect of the calyx clearly relate it to the genus *Otomeria*”). Given that *Otomeria* is now placed in Knoxieae, Francis Hallé’s remark points to a link between *Sacosperma* and that tribe. In the "Flore du Gabon", Nicolas Hallé maintained his brother’s perspective, placing *Sacosperma* in “Hedyotideae” as a transitional genus toward Mussaendeae (Hallé 1966). He also provided the first illustration of the genus (Fig. 1).

The view of the Hallé brothers was not adopted, and subsequent authors included *Sacosperma* in “Hedyotideae” (Robbrecht 1988, 1993) and later in an enlarged tribe Spermacoceae (Groeninckx et al. 2009; Razafimandimbison and Rydin 2024), without further explanation.

### Rhodopentas

The history of *Rhodopentas* begins with Verdcourt’s (1953) revision of the African genus *Pentas* Benth. Faced with the substantial morphological variation within this large genus, Verdcourt recognized six subgenera and further subdivided subg. *Pentas* into five sections. He acknowledged that his groupings “would doubtless be considered of generic standing by many workers, but such disagreement … is unavoidable and of little importance.” He maintained this classification in subsequent flora treatments (Verdcourt 1976, 1989).

A molecular phylogenetic study (Kårehed and Bremer 2007) later revealed *Pentas* to be highly polyphyletic, with clades that aligned closely with Verdcourt’s subgeneric groupings. In light of these findings, the authors recognized most of these clades as distinct genera. One of these, *Pentas* subg. *Pentas* sect. *Coccineae* Verdc., which is morphologically well characterized (Verdcourt 1953, 1976, 1989), was raised to the rank of genus under the name *Rhodopentas*. Verdcourt indeed regarded the sect. *Coccineae* as a particularly well-defined group, and he already noted a close affinity with the genus *Otomeria* because of similarities with *O. elatior* (A.Rich.) Verdc. (flower color), *O. micrantha* K.Schum. (fruits), and *O. volubilis* (K.Schum.) Verdc. (tendency toward a lianescent habit).

## Materials and methods

### Molecular analysis

A multilocus approach was used to investigate the phylogenetic position of *Sacosperma* within Rubiaceae. Leaf tissue samples of four specimens of *Sacosperma paniculatum* were retrieved from the BR herbarium. From these, genomic DNA was extracted using an optimized CTAB protocol (Doyle and Doyle 1987). Polymerase chain reaction (PCR) amplification of the nuclear ribosomal marker internal transcribed spacer (ITS) and the plastid markers (*trnL-F, rps16*) was done with the primers and protocols of Kårehed and Bremer (2007) and performed using a GeneAmp PCR system 9700 (Applied Biosystems). Sequencing reactions were carried out by Macrogen, Inc. (Amsterdam, the Netherlands).

The sequences obtained were edited and assembled in Geneious Prime 2025.0.3 (Biomatters, New Zealand). All previously published sequence data from Knoxieae representatives were obtained from other studies (Rova et al. 2002; Alejandro et al. 2005; Kårehed and Bremer 2007; Kårehed et al. 2008; Groeninckx et al. 2009; Wikström et al. 2010; Krüger et al. 2012; Ferm et al. 2016; Janssens et al. 2016), covering all clades within the tribe. *Danais fragrans* Pers. and representatives of Spermacoceae were used as outgroups. Sequence alignment was performed using MAFFT v.1.5 (Katoh et al. 2002) with the E-INS-i algorithm, a 100PAM/k=2 scoring matrix, a 1.3 gap open penalty, and a 0.123 offset value. The automatically aligned dataset was manually optimized by carefully assessing the homology of every nucleotide at each position in the alignment. The best-fitting nucleotide substitution model for each dataset was selected using jModelTest v.2.1.4 (Posada 2008) under the Akaike information criterion (AIC). The GTR+G model was optimal for ITS, whereas the GTR+I+G model was selected for *rps16* and *trnL-F*.

Phylogenetic conflicts between data matrices were assessed by visually identifying potential conflicting relationships with bootstrap support (BS) values ≥ 70% (Johnson and Soltis 1998; Pirie 2015). Maximum likelihood (ML) trees were inferred using the RAxML search algorithm (Stamatakis et al. 2005) under the GTRGAMMA approximation for ITS and GTRGAMMAI for *rps16* and *trnL-F*. Since no significant topological conflicts were detected, a concatenated DNA matrix was used for Bayesian inference (BI) analysis with BEAST v.1.10 (Suchard et al. 2018). A mixed-model approach was applied, partitioning the datasets to assign distinct evolutionary models to each region (Ronquist and Huelsenbeck 2003). The analysis was run for 10 million generations, with trees sampled every 2,500 generations. A maximum clade credibility tree with a posterior probability limit of 0.5 was reconstructed using TreeAnnotator v.1.10.1 (Suchard et al. 2018). Convergence and effective sample size (ESS) parameters were assessed using TRACER v.1.7.1 (Rambaut et al. 2018). Bayesian posterior probability (PP) values between 0.50 and 0.95 were considered weakly supported, whereas values ≥ 0.95 were considered strongly supported (Suzuki et al. 2002; Alfaro et al. 2003).

A list of all taxa, their voucher information, and GenBank accession numbers is provided in Suppl. material 1.

### Morphological analysis

Morphological information is based on observations of herbarium specimens, field observations by Olivier Lachenaud, and data from two unpublished master’s theses (Dessein 1998; Scheltens 1998). This information was checked against organographical descriptions (*Sacosperma*: Bremekamp 1952; Hallé 1966; *Rhodopentas*: Verdcourt 1976, 1989). When citing specimens in the text, their barcodes are also mentioned to allow easy access to their metadata online. Terminology follows Robbrecht (1988).

Details about the specimens used for morphological investigation can be found in Suppl. material 2. Pollen was observed with a scanning electron microscope at Meise Botanic Garden without being acetolyzed. Wood anatomical sections were prepared based on standard protocols (Jansen et al. 1998). Three wood samples were directly cut with a sliding microtome (GLS1, WSL, Birmensdorf, Switzerland) to obtain ca. 20 µm thick sections. Two samples (*S. paniculatum*, *Louis 13166*, BR0000019729114, and *R. bussei*, *Lovett & Kayombo 4376*, BR0000006743055), however, were too soft and were therefore embedded in paraffin. This involved stepwise soaking of the samples in t-butanol with an increasing concentration for at least 10 h and then immersing them in paraffin for 24 h. The paraffin samples were then cut with a rotary microtome (Leitz, Wetzlar, Germany). Sections were stained with safranin and Astrablue and stepwise dried with 30%, 70%, and 96% ethanol. Finally, sections were cleared with NeoClear (Merck, Darmstadt, Germany), mounted with NeoMount (Merck, Darmstadt, Germany), and left to dry for at least 48 h. The samples were analyzed using a transmission light microscope (Axio Scope.A1, Carl Zeiss Microscopy GmbH, Oberkochen, Germany).

### Biogeographical and climate analyses

Georeferenced occurrence records were downloaded from GBIF for *Sacosperma* (GBIF 2025a) and *Rhodopentas* (GBIF 2025b) and supplemented with data from the literature (Suppl. material 3). Only records based on preserved specimens were considered. Duplicate specimens, identical coordinates, and erroneous records were removed. Aberrant coordinates (e.g., in the ocean) were corrected using locality information. The map was produced in R v.4.5.2 with ggplot2 v.4.0.0 (Wickham 2016) using map tiles by Stadia Maps (https://stadiamaps.com/) and OpenMapTiles (https://openmaptiles.org/) and data by OpenStreetMap (https://www.openstreetmap.org). The obtained distribution ranges of both genera were compared with country-level data from the African Herbaria Community Portal (AHCP 2025), the African Plant Database (APD 2025), and Plants of the World Online (POWO 2025). We follow Linder et al. (2012) for the delimitation of the phytochoria.

The yearly patterns of monthly precipitation data for the period 1981–2010 (CHELSA v.2.1; Karger et al. 2017) were compared between *Sacosperma* and *Rhodopentas*. The boxplots were prepared using the R packages terra v.1.8-70 (Hijmans 2025), ggplot2 v.4.0.0 (Wickham 2016), and dplyr v.1.1.4 (Wickham et al. 2023).

## Results and discussion

### Molecular evidence for the inclusion of *Sacosperma* in Knoxieae

**Figure 2.**
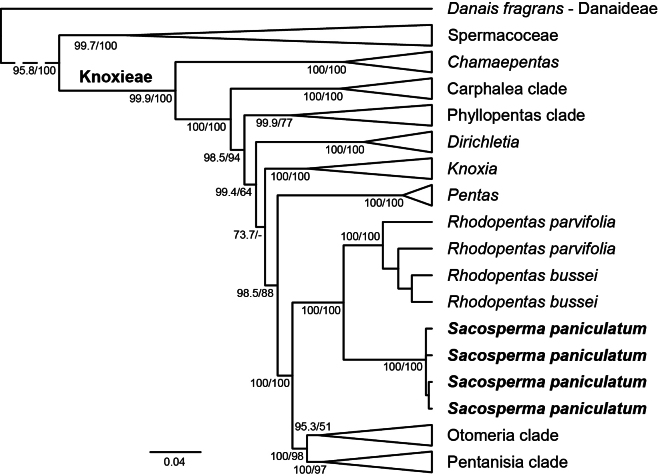
Modified phylogenetic tree of Knoxieae based on the Bayesian analysis showing that the genus *Sacosperma* (bold) is nested within the tribe and is sister to *Rhodopentas*. Posterior probabilities and bootstrap values are indicated at the nodes.

The tribe Knoxieae (subfamily Rubioideae, Spermacoceae alliance; Razafimandimbison and Rydin 2024) forms a well-supported monophyletic group, and its major clades are similar to those of Kårehed and Bremer (2007) (Fig. 2; Suppl. materials 4, 5). This consistency is expected, given that most of the sequence data used here were derived from that study. The genus *Chamaepentas* Bremek. sits as sister to the rest of the tribe. The next node splits off the Carphalea clade, with the genera *Carphalea* Juss., *Paracarphalea* Razafim., Ferm, B.Bremer & Kårehed, and *Triainolepis* Hook.f., from the remaining Knoxieae. The next clade, the Phyllopentas clade, contains *Dolichopentas* Kårehed & B.Bremer, *Parapentas* Bremek., and *Phyllopentas* (Verdc.) Kårehed & B.Bremer. The genera *Dirichletia* Klotzsch and *Knoxia* L. are monophyletic, but their relationships to the remaining genera of Knoxieae are unresolved (PP 99.4/BS 64 and PP 73.7/BS –, respectively). The next clade consists of the genus *Pentas* sensu stricto, which is monophyletic. *Sacosperma paniculatum* is nested within Knoxieae and is sister to *Rhodopentas*. Both are sisters to the remainder of the tribe, consisting of two clades: the *Otomeria* clade, including *Batopedina* Verdc., *Otiophora* Zucc., *Otomeria*, and *Parapentas setigera* (Hiern) Verdc., and the Pentanisia clade, including *Pentanisia* Harv. and close relatives.

This study provides the first sequence data for the genus *Sacosperma* and unambiguously supports its transfer from Spermacoceae to Knoxieae, firmly rejecting alternative hypotheses such as Hallé's (1961) suggestion that it belongs to tribe Mussaendeae (now in subfamily Dialypetalanthoideae). *Sacosperma* is sister to the small genus *Rhodopentas*. The two have never been regarded as closely related, and in the only detailed morphological study that mentioned both taxa (Verdcourt 1953), no direct comparison was made. We address this gap by comparing the two taxa.

### Morphological overview of *Sacosperma*, compared to the sister genus *Rhodopentas*

Our observations of *Sacosperma* mainly relate to *S. paniculatum*; the second species, *S. parviflorum*, is much less collected but generally very similar.

#### Habit

**Figure 3.**
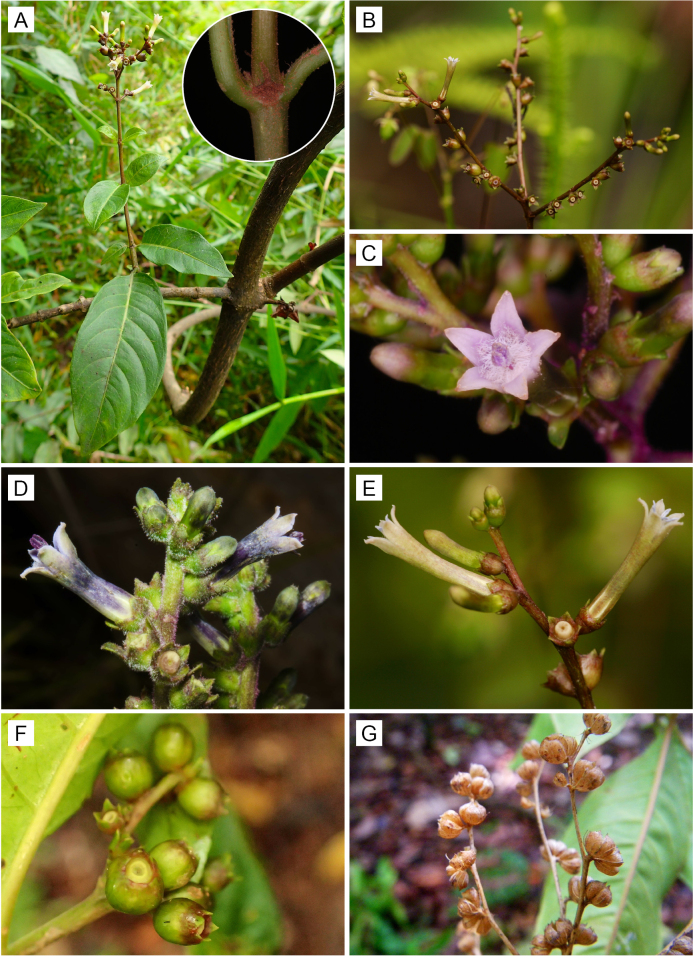
Morphology of *Sacosperma paniculatum*. **A**. Winding main stem and lateral twig with anisophyllous leaves and a terminal inflorescence. The inset shows an unidentate stipule. **B**. Terminal inflorescence with flower pairs in helicoidal arrangement on brown-colored axes. **C**. Inflorescence with purplish axis and pentamerous, longistylous flower with purplish corolla. **D**. Inflorescence with green calyces and bluish and whitish corollas. **E**. Inflorescence with brown-colored axes and paired flowers with pale greenish, whitish corollas and reddish-brown calyces. Note the slits at the base of the corollas. **F**. Infructescence with young, green fruits. **G**. Infructescence with dry, dehiscent capsules. Photos by Nicolas Texier (A, *Texier 2268*; F, *Texier 178*); Steven Dessein (B, E, *Dessein 1750*); Ehoarn Bidault (C, *Bidault 149*; D, *Bidault 6002*); and Olivier Lachenaud (G, *Lachenaud 1628*).

*Sacosperma* species are woody lianas (Fig. 3A). While typically reaching only a few meters in height, they have been reported to climb up to 12 m, with stems as thick as a human thumb (e.g., *S. paniculatum*, *Louis 12790*, BR0000006822910). Their lateral twigs, which are typically positioned at approximately right angles to the main stem, are relatively short, generally comprising only 3–6 internodes, and bear a terminal inflorescence (Fig. 1).

The growth habits of Knoxieae are highly diverse, ranging from small trees and shrubs to small annual herbs. In this regard, *Sacosperma* appears to be the tallest representative of Knoxieae. Only a few members of Knoxieae share the climbing habit of *Sacosperma*. Notably, *Otomeria volubilis* is a winding liana that can become entangled in the surrounding vegetation and reach heights of over 5 m (Hallé 1966). The sister genus of *Sacosperma*, *Rhodopentas*, a genus of (sub)shrubs, tends to be winding and is noted by some collectors as lianescent (e.g., *R. bussei* (K.Krause) Kårehed & B.Bremer, *Mwangoka & Maingo 1300*, BR0000009269330).

**Figure 4.**
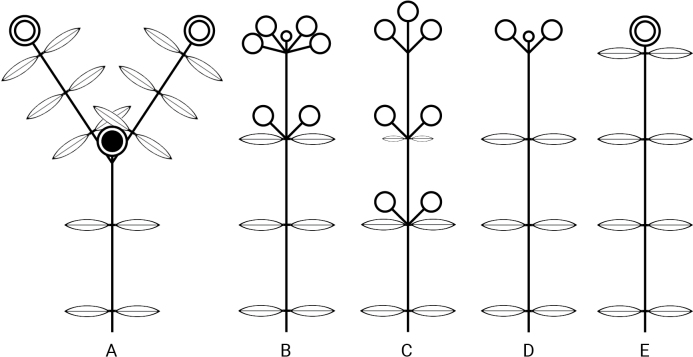
Schematic representation of the inflorescences of some Knoxieae. **A**. *Rhodopentas bussei*. **B**. *Sacosperma paniculatum*. **C**. *Knoxia manika*. **D**. *Pentanisia schweinfurthii*. **E**. *Neopentanisia gossweileri*. Double circles = inflorescences; single circles = partial inflorescences, drawn small in case of reduction; filled circle = infructescence of previous growing season. Anisophylly and leaf size variation are not depicted.

*Sacosperma* is climbing by means of winding or twining stems, but further details on its architecture could not be deduced from herbarium specimens, so field studies are needed. However, the architecture of *Rhodopentas* is more evident from a number of herbarium specimens (e.g., *R. bussei, Bridson 120*, BR0000017774642). The flowering twigs, which terminate in a compact inflorescence, exhibit a dichotomous branching pattern. Their growth is arrested by the development of the infructescence, after which the twigs reiterate from two opposite axillary buds located just below the infructescence (Fig. 4A). Occasionally, only one of these buds develops, resulting in a unilateral reiteration (e.g., *R. bussei*, *Kayombo & Ntemi Sallu 3006*, BR0000009374997).

#### Wood anatomy

The stems of *Sacosperma* are hollow, which is unusual in Knoxieae, whereas those of *Rhodopentas* are solid.

The two wood samples of *Sacosperma paniculatum* studied show mainly solitary vessels without distinct growth rings. Pits on radial and tangential walls of imperforate tracheary elements are distinctly bordered, indicating the presence of fiber-tracheids, while tracheids also occur. *Rhodopentas*, however, has more pronounced growth rings, with vessels either solitary or grouped in radial patterns, and a tendency toward dendritic vessel arrangement. Vessel walls of *Rhodopentas* are thicker than those in *Sacosperma*. *Rhodopentas* also differs from *Sacosperma* by the presence of libriform fibers with reduced pit borders on radial and tangential walls. Tracheids are common and difficult to distinguish from fibriform vessels, which may have very small, simple perforation plates.

Shared features of both genera include the lack of axial parenchyma and the presence of narrow, uniseriate to biseriate rays. The bordered pits of both genera are minutely vestured, and vessel-parenchyma pitting shows reduced pit borders. Raphides occur in the pith tissue of *Rhodopentas*.

Following Jansen et al. (2002), the combination of fiber-tracheids and solitary vessels in *Sacosperma* is characteristic of wood type I, while the libriform fibers and arrangement of vessels in radial multiples in *Rhodopentas* refer to wood type II. The difference in growth rings between both genera likely reflects annual rainfall patterns. While type I is the most common wood type within Rubioideae, including representatives of “Hedyotideae,” type II appears to have evolved in taxa that are semi-woody to herbaceous (Jansen et al. 2002). Knoxieae includes mainly wood type I, as reported for *Otiophora*, *Otomeria*, *Pentas*, *Pentanisia*, and *Phyllopentas* (Jansen et al. 2002; Lens et al. 2009). However, the radial arrangement of vessel multiples in *Carphalea* and *Sacosperma*, and the pronounced vessel grouping in *Dirichletia* and *Triainolepis*, likely reflect an adaptation to seasonal drought (Lens et al. 2009). The presence of two different wood types within Knoxieae appears to be related to how woodiness evolved (primary vs. secondary woodiness) and is likely affected by seasonality in rainfall patterns. Therefore, the wood anatomy of *Sacosperma* offers no arguments against its placement in Knoxieae.

#### Aluminum accumulation

Aluminum accumulation is reported to occur in leaves of three out of four specimens of *S. paniculatum* (Jansen et al. 2000), but its presence is unknown for *Rhodopentas*. It is generally more common within Knoxieae than Spermacoceae, with aluminum accumulation being present in 42 out of 60 taxa (i.e., 70%) for Knoxieae and 23 out of 62 taxa (i.e., 36%) for Spermacoceae (Jansen et al. 2000). However, these numbers should be interpreted carefully, as more material should be tested. So far, aluminum accumulation does not provide additional evidence for or against the transfer of *Sacosperma* to Knoxieae.

#### Leaves and stipules

*Sacosperma* has opposite, petiolate leaves with elliptic, ovate to narrowly ovate leaf blades with an attenuate or acuminate apex and an attenuate base. Anisophyllous nodes frequently occur (e.g., *S. paniculatum*, *Jongkind 11937*, BR0000019728308), especially at the base of flowering twigs (Fig. 3A). Hallé (1966) did not mention anisophylly in his description, although he depicted a lateral flowering branch with two strongly anisophyllous proximal nodes (Fig. 1). *Sacosperma* has persistent interpetiolar stipules that are unidentate (Fig. 3A) or bidentate, sometimes with colleters forming additional smaller teeth. The underside of the leaf blades in *S. paniculatum* varies from nearly glabrous to covered with erect, crisped, multicellular hairs, especially on the nerves. In the latter case, the hairs are sometimes denser in the nerve axils and tend to form tuft domatia. In the more glabrous variants, pocket domatia or domatia intermediate between the pocket and pit types may occur. Hallé (1966) was the first to report the occurrence of domatia in *Sacosperma*. His description (“domaties axillaires pubescentes à ouverture ± apparente”) implies that they are consistently present and, due to the presence of an opening, belong to the crypt or pit type, but as mentioned above, *S. paniculatum* is variable with regard to the presence and type of domatia.

**Figure 5.**
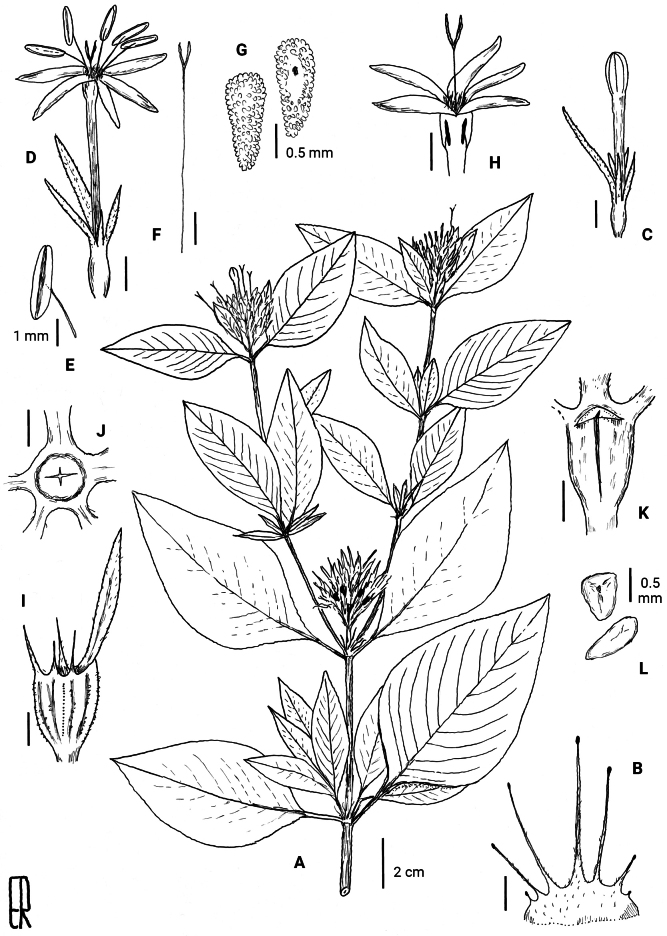
*Rhodopentas bussei*. **A**. Dichotomous flowering twig, with infructescence from the previous growing period. **B**. Stipule with setae and colleters. **C**. Flower bud, note the unequal calyx lobes. **D**. Open medistylous flower. **E**. Stamen (from D). **F**. Style and stigma (from D). **G**. Two views of a placenta. **H**. Top of open longistylous flower (silhouettes show stamen position in widened top of corolla tube). **I**. Fruit, dotted line is where mericarps separate. **J**. Fruit seen from above, from inside to outside: beak with four slits, scar of corolla insertion, calyx tube, and bases of calyx lobes. **K**. Artificially removed half of the fruit, showing beak and septum with loculicidal slit. **L**. Seeds. All scale bars are 2 mm, except when indicated otherwise. Drawn by Elmar Robbrecht based on *Mwangoka & Iginasi 2838* (BR0000009527577) (**A**), *Rajabu Hizza 97* (BR0000009178625) (**B**), *Lewalle 4735* (BR0000009812697) (**C–G**), *Richards 4807* (BR0000017774963) (**H**), and *Kibure 33* (BR0000009256538) (**I–L**).

*Rhodopentas* also has opposite, petiolate leaves but often appears to be verticillate due to the development of axillary short shoots bearing leaves somewhat smaller than those on the main stems. Such short axillary shoots are not observed in *Sacosperma*. Nodes in *Rhodopentas* are frequently strongly anisophyllous (e.g., *R. bussei*, *Drummond & Hemsley 3813*, BR0000017774574). *Rhodopentas* has divided stipules with 3–9 linear setae crowned by a single colleter (Fig. 5B), but such stipules are standard in Knoxieae. Domatia are absent in *Rhodopentas*, as in all other Knoxieae.

#### Inflorescences

The inflorescences of *Sacosperma* are terminal on relatively short lateral twigs and mostly frondobracteose (i.e., with gradual transition from leaves to bracts) (Figs 1, 3A, 4B). The proximal part is mostly provided with well-developed leaves, only slightly reduced compared to the leaves of the vegetative region, whereas bracts in the distal parts are reduced or even absent. The inflorescence is divided into partial inflorescences (PIs) in a quite variable way. In the short shoot depicted in Fig. 1 (schematically represented in Fig. 4B), one can distinguish, from base to top, a vegetative region with (i) two anisophyllous nodes and (ii) one node with normal leaves, and a reproductive region with (iii) a pair of PIs in the axils of slightly reduced leaves, (iv) two pairs of axillary PIs in an apparent coaxillary position, and (v) a strongly reduced terminal PI. Many variants of these patterns can be observed, however, with a common trend: the axillary PIs tend to be perpendicular to the main axis. For the structure of (iv), two explanations are plausible: coaxillary primordia or extreme reduction of the internode between the two pairs. Since bracts are absent in this zone of the inflorescence, there is no definitive proof for either hypothesis. The PIs are generally elongated and spike-like (Fig. 1); the flowers are in pairs (Fig. 1), and these are helicoidally arranged (Fig. 3B, E). The color of the axes of the inflorescences can be purplish, reddish, or brown (Fig. 3B, C, E).

Inflorescences of Knoxieae are usually terminal (e.g., Fig. 4). In many representatives, the reproductive region is strictly separated from the vegetative region. This is the case in *Rhodopentas*, which has rather compact terminal frondobracteose inflorescences, mostly composed of three equal-sized PIs (Fig. 4A). Only the first-order branching of the inflorescence clearly reflects the basic thyrso-paniculate structure. In the upper PI, congestion is so strong that the basic structure can no longer be discerned.

Inflorescence morphology of the tribe Knoxieae is diverse. The following trends are postulated: (i) inflorescences become less compact, with the terminal inflorescence dividing into several PIs. In *Pentanisia schweinfurthii* Hiern, the inflorescence axis is closed by a terminal reduced PI, and the development of two axillary PIs is promoted (Fig. 4D). In *Knoxia manika* (Verdc.) Puff & Robbr., 1–3 pairs of opposite axillary PIs are positioned below the terminal PI; the inflorescence is frondobracteose, with the two lower PI pairs in the axils of slightly reduced leaves (Fig. 4C). (ii) Inflorescence axes elongate to become spicate (often in the stage of fruit maturation), with two flowers per node in a helicoidal arrangement (e.g., *Otomeria guineensis* Benth.; Hallé 1966: fig. 20-9). This was discussed in detail for *Otiophora* by Puff (1983: fig. 1), who provided an explanation for how this type of PI can be derived from the thyrso-paniculate structure, which is the basic inflorescence type in Rubiaceae. It results from the unequal promotion of lateral elements of the inflorescence, such as PIs, followed by the alternating helicoidal arrangement of the promoted PIs.

Despite being highly derived, inflorescences of *Sacosperma* (Figs 1, 4B) fit well with patterns observed in Knoxieae and show the above trends: (i) closure by a reduced terminal PI and (ii) spike-like PIs with flower pairs in a helicoidal arrangement. The inflorescences of *Sacosperma* closely resemble those of some *Bertiera* species (Bertiereae, Dialypetalanthoideae), in particular *B. breviflora* Hiern and *B. aequatorialis* N.Hallé. In this genus, the basic inflorescence type is thyrso-paniculate, but several modifications occur (Robbrecht et al. 1993), including congestion of PIs resulting in spike-like inflorescences, such as depicted by Hallé (1960: fig. 1c). This is a case of parallel evolution.

#### Flowers

##### Merosity

The flowers of *Sacosperma* are 5-merous (Fig. 3C) and herein match the majority of Knoxieae (including *Rhodopentas*), which have 5(–6)-merous flowers, though 4-merous flowers do occur, e.g., in *Knoxia*, the former monospecific genus *Paraknoxia* (now *Pentanisia parvifolia* Stapf ex Verdc.), and in some species of *Carphalea*, *Dirichletia*, and *Paracarphalea*.

In contrast, Spermacoceae has predominantly 4-merous flowers. The 5-merous genus *Pentodon* Hochst. is the sole tropical African exception in the tribe.

##### Flower dimorphism

Bremekamp (1952) was the first to report heterodistyly in *Sacosperma*, with longistylous flowers having included anthers (Fig. 1) and brevistylous ones having exserted anthers (so-called complete heterodistyly, Robbrecht 1988: 124).

The same two floral morphs occur in *Rhodopentas*, but the stamens (in brevistylous flowers) or styles (in longistylous flowers) are more exserted than in *Sacosperma*. Furthermore, the genus is heterotristylous: next to the longi- (Fig. 5H) and brevistylous morphs, there are specimens with a medistylous morph. It is very similar to the brevistylous morph, but the stigmatic lobes just surpass the bearded corolla throat and are positioned between the filaments (Fig. 5D). In the treatment of *Pentas* sect. *Coccineae* (= *Rhodopentas*), Verdcourt (1953) described Pentas (Rhodopentas) bussei as dimorphic and Pentas (Rhodopentas) parvifolia as trimorphic, but we observed specimens with the medistylous morph in *R. bussei* as well. In later publications, Verdcourt (1976, 1989) described the flowers of sect. *Coccineae* as dimorphic.

Heterodistyly is the ancestral state in the Spermacoceae alliance and the default situation in Knoxieae, with rare reversals to monomorphic flowers, e.g., in some species of *Pentas* (Verdcourt 1953). Reversals to monomorphism occurred more frequently in Spermacoceae (Ferrero et al. 2012). Heterotristyly is a rare condition in Rubiaceae, e.g., in *Psychotria cyanopharynx* K.Schum. and *P. oblanceolata* (R.D.Good) Ruhsam (Lachenaud 2019), and in some species of *Eumachia* DC. (Lachenaud in press).

##### Calyx

The calyx lobes in *Sacosperma* are often (though not always) slightly unequal; occasionally, one of them may become leafy (Hallé 1966). The color of the calyx is variable and can be reddish-brown, bluish-green, or green (Fig. 3C, D, E).

This matches *Rhodopentas* (Fig. 5C, D, I) and other Knoxieae, which almost universally exhibit unequal calyx lobes. This character is especially prominent in *Carphalea*, *Paracarphalea*, and *Phyllopentas*, where the greatly enlarged calyx lobes are colored and function as semaphylls.

##### Corolla

Flowers of *Sacosperma* have small corollas that are only 7–9 mm long (tube and lobes), with acute lobes that are hardly longer than 1 mm (Hallé 1966). The genus is remarkable in having corollas that are highly variable in color; even on a single flower, the base and top may have different colors (e.g., *S. paniculatum*, Fig. 3D and *Louis 13166*, BR0000006809539). The corollas of mature flowers are generally pale: whitish, purplish, bluish, or reddish brown (Fig. 3C, D, E). The outside of the corolla is glabrous, while the inside is villose in the upper half of the tube and puberulous near its base, with a glabrous area between.

Members of Knoxieae mostly have medium-sized to relatively large corollas; for example, *Rhodopentas* species have tubes of 7–22 mm long and lobes of 2.5–10 mm long (Verdcourt 1976). Minute corollas (< 10 mm long, as in *Sacosperma*) are rarer but are reported, for example, in *Otomeria micrantha* K.Schum. (Hallé 1966) and *Carphalea cloiselii* Homolle (Puff 1988). Blue or purple colors (sometimes vibrant) are predominant in Knoxieae, while *Rhodopentas* has bright red corollas (Verdcourt 1953).

##### Fenestrated corollas and floral ontogeny

Vrijdaghs et al. (2015) examined the floral ontogeny of genera of the Spermacoceae alliance reported to have fenestrated corollas, viz., *Paederia* L. (*P. farinosa* (Baker) Puff and *P. thouarsiana* Baill.) in Paederieae, *Pentodon* (*P. pentandrus* Vatke) and *Spermacoce* L. (*S. occultiseta* Harwood) in Spermacoceae, and *Pentas* (*P. lanceolata* (Forssk.) Deflers) and *Sacosperma* (*S. paniculatum*) in Knoxieae (Fig. 3E). They showed that corolla splits appear actively in the basal part of an initially closed tubular corolla in *Pentas* and *Sacosperma* (Knoxieae). In contrast, the corolla splits result from incomplete fusion of the corolla lobes in *Pentodon* (Spermacoceae). In addition, general floral development in *Sacosperma paniculatum* and *Pentas lanceolata* was found to be very similar.

The corollas of *Rhodopentas* occasionally show basal splits as well (e.g., *R. bussei*, *Schlieben 1609*, BR0000019800028, *Polhill & Paulo 678*, BR0000017774659), although their occurrence is not constant.

##### Gynoecium

*Sacosperma* and *Rhodopentas* have 2-carpellate ovaries. The placentas are peltate—hence the original name *Peltospermum* (Verstraete et al. 2025)—and covered with numerous ovules. The style is bilobed.

The same placentation type is found in other multiovulate Knoxieae and, by extension, Rubiaceae. The number of carpels in Knoxieae is variable, often two, sometimes 3–5 (*Pentanisia* pro parte) or more (4–10 in *Triainolepis*).

#### Fruits

The fruits of *Sacosperma* and *Rhodopentas* show many similarities. Both have pale brown, glossy, longitudinally ribbed capsules, with a weakly developed beak (an outgrowth of the endocarp; see Buchner and Puff 1993) that remains hidden in the calyx tube. The capsules eventually split into two valves that remain attached to the stalk.

*Sacosperma* capsules (Fig. 3G) are ± spherical or rarely obovoid (e.g., *Dessein et al. 1687*, BR0000013168735) and diplophragmous, i.e., they exhibit deep primary septicidal dehiscence and shallow secondary loculicidal dehiscence. Dispersal of the seeds occurs by disintegration of the thin dissepiment between the diverging valves.

*Rhodopentas* capsules (Fig. 5I, J) are slightly obconical; the beak at maturity shows two septicidal and two short loculicidal slits. The dissepiment is more coriaceous than in *Sacosperma*, and the slit of the beak continues on the dissepiment side of the valve (Fig. 5K).

Multiovulate Knoxieae all have capsules, with great variation in patterns of dehiscence. The large diversity of fruits in uniovulate Knoxieae is documented in detail by Puff and Robbrecht (1989).

#### Seeds

**Figure 6.**
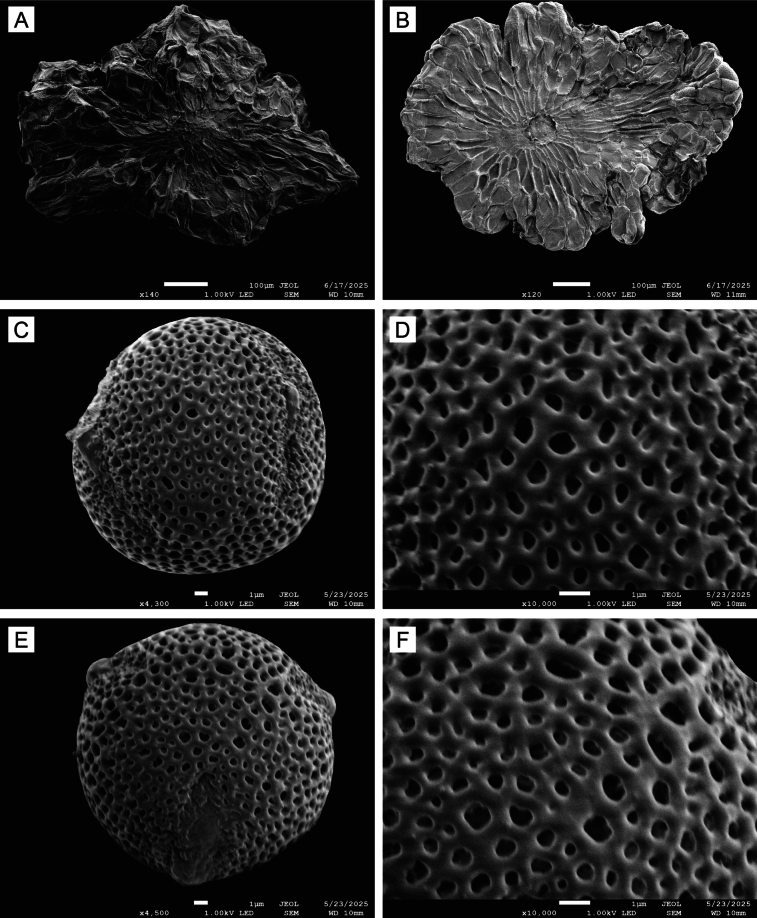
Seeds and pollen of *Sacosperma paniculatum*. **A**. Abaxial side of a seed. **B**. Adaxial side of a seed showing exotestal cells with a reticulate pattern and hilum. **C**. Equatorial view of a pollen grain from a brevistylous flower. **D**. Mesocolpium showing the microreticulate tectum. **E**. Polar view of a pollen grain from a brevistylous flower. **F**. Apocolpium showing the perforate tectum. **A**. From *Bos 3131* (BR0000019728582); **B**. From *Louis 13851* (BR0000019729121); and **C–F**. from *De Koning 993* (BR0000019728391).

*Sacosperma* has brown, dorsiventrally flattened, polygonal seeds with a slightly eccentric hilum (Fig. 6A, B). Bremekamp (1952: 43) explicitly stated that the seeds of *Sacosperma* are not winged and that Bentham (1849) was incorrect due to having examined immature material. However, there is a small part of the seed coat surrounding the seed that does not cover the endosperm, so the seeds are slightly winged. The illustration by Hallé (1966; Fig. 1) of a marginal wing is somewhat exaggerated. The exotestal cells are polygonal, somewhat elongated, with straight radial walls. The inner tangential walls have reticulate thickenings, while the outer tangential walls are punctulate.

*Rhodopentas* seeds have the same shape but are wingless (Fig. 5L). The exotestal cells are elongated and have a wavy radial wall, while the outer tangential wall shows a delicate punctate pattern (Dessein 1998: fig. 29A–D).

#### Pollen

*Sacosperma* has ± spheroidal, 3-colporate pollen grains with a microreticulate tectum, with perforations 0.1–1.2 µm in diameter (Fig. 6C–F). We observed no noticeable differences between pollen of the brevi- and longistylous morphs.

*Rhodopentas* has subprolate, 3-colporate pollen with a perforate tectum (Dessein et al. 2000: figs 10, 22). Knoxieae are relatively stenopalynous: they are almost universally 3-colporate (as is the case for many Rubiaceae groups) and show variation mainly regarding exine patterns. Spermacoceae, in contrast, are strongly eurypalynous and often have a higher number of apertures (e.g., Dessein et al. 2002). Pollen morphology, particularly the perforate exine, supports the sister relationship of the two genera, and the 3-colporate condition agrees with their inclusion in Knoxieae.

### Distribution, habitat, and climate preference of *Sacosperma* and *Rhodopentas*

#### Distribution of *Sacosperma*

The genus *Sacosperma* occurs in West Tropical and Central Africa and represents a mainly Congolian element distributed across the Guinea, Congo, and Shaba subregions (sensu Linder et al. 2012). It also radiates into the Sudanian Region, notably in the Central African Republic, northern Nigeria, northern Ivory Coast, Guinea, Guinea-Bissau, and southern Senegal. Under White’s (1983) system, this Sudanian extension primarily falls within the Guineo-Congolia/Sudania Transition Zone.

**Figure 7.**
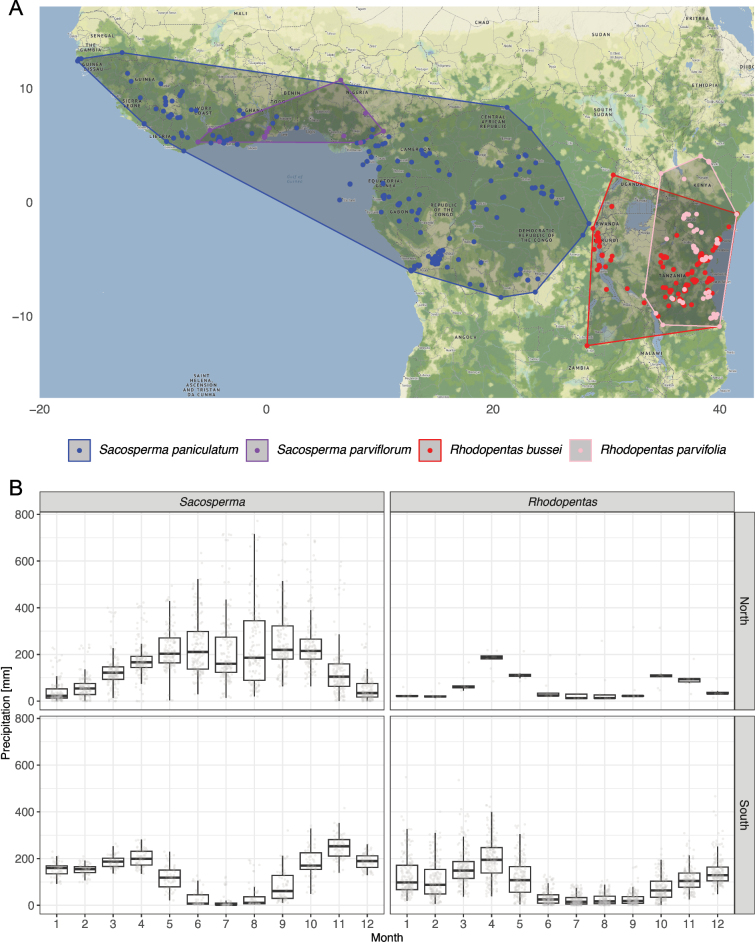
**A**. Distribution of *Sacosperma* (blue, purple) and its sister genus *Rhodopentas* (red, pink) in tropical Africa. The polygons represent the smallest areas enclosing known coordinates of each species. **B**. *Sacosperma* occurs mostly in humid environments (north of the equator: n = 145; south of the equator: n = 63), while *Rhodopentas* is found in regions with a pronounced dry season (north of the equator: n = 10; south of the equator: n = 181).

The most widespread species, *Sacosperma paniculatum*, determines the overall distribution of the genus: northern Angola, Benin, Cameroon, Central African Republic, Democratic Republic of the Congo, Equatorial Guinea, Gabon, Ghana, Guinea, Guinea-Bissau, Ivory Coast, Liberia, Nigeria, Republic of the Congo, São Tomé and Príncipe, southern Senegal, Sierra Leone, and Togo (Fig. 7A). The syntype *Heudelot 628* (K00414245) is noted as from “Senegambia,” and although interpreted as from Gambia by Kew, the "Flora of West Tropical Africa" (FWTA) interpretation of Guinea is more plausible. This is supported by the P duplicate (P04478256), which bears the locality “Rio Nunez,” and contemporary collections of other species such as *Heudelot 626* (P00088326) and *Heudelot 638* (P00413085) from Guinea. Our findings regarding the distribution pattern of the species correspond with what is found in the literature (Bentham 1849: 400; Hiern 1877: 53; Taylor 1944: 219; Hepper and Keay 1963: 213; Hallé 1966: 121; Akoègninou et al. 2006) and in online databases (AHCP 2025; APD 2025; GBIF 2025a; POWO 2025).

*Sacosperma parviflorum* is more restricted, found only in West Tropical Africa and confined to the Guinea subregion of the Congolian Region: western Cameroon, Ghana, Ivory Coast, and Nigeria (Fig. 7A). A Tanzanian specimen (*Mlangwa & Mbuso 1309*, MA-01-00766797) labeled as *Pentas parviflora* Benth. was excluded due to misidentification, referring to *Pentas parvifolia* Hiern (= *Rhodopentas parvifolia* (Hiern) Kårehed & B.Bremer) (see *Mlangwa & Mbuso 1309*, MO-2293002). The literature (Taylor 1944: 219 footnote; Hepper and Keay 1963: 213) and online databases (AHCP 2025; APD 2025; GBIF 2025a; POWO 2025) agree with this distribution.

#### Distribution of *Rhodopentas*

*Rhodopentas bussei* is distributed across Burundi, the Democratic Republic of the Congo, Kenya, Malawi, northern Mozambique, Somalia, Tanzania, Uganda, and Zambia (Fig. 7A). Records from Malawi (*Buchanan 1187*, US02361463, and *Buchanan 1285*, US02361462) are not shown on the map due to the lack of locality data (Verdcourt 1953). *Rhodopentas parvifolia* occurs in southern Ethiopia, Kenya, northern Mozambique, southern Somalia, Tanzania, and eastern Uganda (Fig. 7A).

The genus *Rhodopentas* occurs in East Africa, and its species are mainly found in the seasonally dry Zambezian Region, with extensions into the arid Somalian Region and the transitional Shaba subregion. The distributions of *Sacosperma* and *Rhodopentas* come close to each other in the Albertine Rift in eastern DR Congo.

### Habitat and climate preference

Both species of *Sacosperma* are heliophilous and occur in rainforest edges. They are especially frequent in humid environments, typically along riverbanks, lake edges, semi-shaded streams, and marshy bush (Hallé 1966), with even a record of *S. paniculatum* from mangroves (*Den Outer-Helder 47*, BR0000019728353), but they may also occur in well-drained areas, including inselberg summits (e.g., *Parmentier 1547*, BR0000009422629). *Rhodopentas* species occur in dry evergreen forest, gallery forest, woodland, wooded grassland, bushland, and thickets, often riverine or along streams, but also occasionally in rocky areas (Verdcourt 1976, 1989).

Climate preferences differ markedly between the two genera (Fig. 7B). *Rhodopentas* occurs in regions with a very strong dry season from June to September, whereas *Sacosperma* inhabits generally more humid climates that show greater variation in seasonality across its wide geographical range. North of the (thermal) equator and along the West African coast, the dry season occurs from December to February, while south of the (thermal) equator and across Central Africa, it occurs between June and September. These patterns are reflected in the boxplots (Fig. 7B): depending on the location of the plants in relation to the equator, *Sacosperma* has dry periods in the months of either June to August or December to February. Consequently, *Sacosperma* occupies a broader climate envelope regarding precipitation than *Rhodopentas*.

### Phytogeographical synthesis

The distribution patterns of *Sacosperma* and *Rhodopentas* correspond well with the range of the tribe Knoxieae, which is found throughout sub-Saharan Africa except for the Kalahari, Namib, and Cape subregions. Knoxieae is more diverse in the seasonally dry areas from the Horn of Africa to the Zambezian Region and southern Africa (Robbrecht 1996) and in Madagascar (Wikström et al. 2010), but some genera, such as *Otomeria* and *Parapentas*, also occupy the wetter, less seasonal Congolian Region (POWO 2025), similar to *Sacosperma*.

The tribe Spermacoceae is pantropical and widespread across sub-Saharan Africa, where its diversity centers in Africa are the same as for Knoxieae (viz., the seasonally dry Zambezian Region and Madagascar). Congolian endemics are rare; one example is *Stephanococcus* Bremek., which, like *Sacosperma*, has a lianescent growth form but is herbaceous (Bremekamp 1952). The genus *Pentodon* shares a similar distribution with *Sacosperma* but extends further east into the Zambezian Region and Madagascar (POWO 2025).

While the distribution of *Sacosperma* is consistent with both Knoxieae and Spermacoceae, the distinct biogeographical patterns and climate preferences of *Sacosperma* and *Rhodopentas* nonetheless support their recognition as two separate genera.

### Two final considerations

#### *Sacosperma* and *Rhodopentas*: monospecific genera?

Our observations reveal considerable morphological variation within *Sacosperma paniculatum*, particularly in leaf indumentum, the presence and type of domatia, and flower color. A thorough revision of the genus would be desirable to assess whether this variation corresponds to infraspecific categories and whether *S. paniculatum* and *S. parviflorum* are distinct species. These two species differ in little but the longer calyx lobes and usually more contracted inflorescences of *S. parviflorum*, and the latter might well be just a variant of *S. paniculatum*.

The taxonomy of *Rhodopentas* also warrants further investigation. Verdcourt (1953) separated the two species (then in *Pentas*) based on vegetative characters such as leaf size and number of secondary veins and observed that they “tend to intergrade”; he described in each species several formae to illustrate their considerable morphological variation. Later, he considered the formae in *P. bussei* “not worth retaining” (Verdcourt 1976, 1989). The sympatric distribution of the two species (Fig. 7A) and our molecular analyses (Fig. 2) suggest that perhaps only one variable species should be recognized. Alternatively, *R. parvifolia*, which shows significant variation, e.g., in the length of the corolla tube (generally shorter in Kenyan than in Tanzanian material), might conceivably include several taxa.

#### A genus pair or a single genus?

Although *Sacosperma* and *Rhodopentas* share a number of morphological characters (Table 1), these are not specific to these two genera and occur in other Knoxieae as well. Moreover, their clear morphological differences (i.e., habit, imperforate tracheary elements, domatia, stipules, inflorescences, flower size and color), as well as their different distribution and climate preference, strongly support maintaining the two genera as distinct. Although we did not perform a molecular dating analysis, the pronounced morphological divergence between the two genera suggests a relatively ancient split, likely followed by adaptation to contrasting ecological conditions.

**Table 1.** Similarities (in bold) and differences between the sister genera *Sacosperma* and *Rhodopentas*.

	* Sacosperma *	* Rhodopentas *
Habit	**liana**	(sub)shrub, sometimes **lianescent**
Stems	hollow	solid
Wood	no growth rings, mainly solitary vessels, fiber-tracheids	indistinct to distinct growth rings, vessels both solitary and in radial multiples, libriform fibers
Leaves	**proximal nodes of flowering twigs often strongly anisophyllous**	
Domatia	usually present	absent
Stipules	uni- or bidentate (sometimes with additional smaller teeth)	divided, numerous colleter-tipped setae
Inflorescences	loose, composed of 3–9 spicate PIs with flower pairs in a helicoidal arrangement, closed by a reduced terminal PI	compact, composed of 3 congested equal PIs
Flowers	**5-merous**, heterodistylous	**5-merous**, heterotristylous
Corolla	small (< 10 mm long), pale: whitish, purplish, bluish, reddish brown	medium-sized (> 10 mm long), bright red
Corolla splits	**present**	sometimes **present**
Calyx lobes	often slightly **unequal**	**unequal**
Ovary	**2-carpellate ovaries with peltate placentas covered by numerous ovules**	
Fruits	**brown, glossy, ribbed capsules separating into two valves**	
Seeds	slightly winged **dust seeds**	wingless **dust seeds**
Radial walls of exotestal cells	straight	wavy
Pollen	**3-colporate pollen with microreticulate tectum**	
Distribution	West and Central Africa; Congolian Region	East Africa; Zambezian Region
Habitat and ecology	rainforests; humid environments with variation in seasonality	gallery forest, dry evergreen forest, woodland, wooded grassland, often riverine; seasonal environments

## Additional information

### Use of AI

No use of AI was reported.

### Author ORCIDs

Brecht Verstraete https://orcid.org/0000-0001-9332-7798

Olivier Lachenaud https://orcid.org/0000-0001-8972-4096

Petra De Block https://orcid.org/0000-0001-7210-7868

Arne Mertens https://orcid.org/0000-0003-3272-9464

Steven B. Janssens https://orcid.org/0000-0003-1246-9474

Judith R. Schepers https://orcid.org/0000-0001-6604-7942

Steven Jansen https://orcid.org/0000-0002-4476-5334

Elmar Robbrecht https://orcid.org/0000-0002-3811-595X

### Data availability

All of the data that support the findings of this study are available in the main text or Supplementary Information.

## Supplementary materials

Supplementary material 1 Specimens used in the molecular analysis, with tribal placement, taxon names, voucher information, specimen links, and GenBank accession numbers Data type csv https://binary.pensoft.net/file/1570139This dataset is made available under the Open Database License (http://opendatacommons.org/licenses/odbl/1.0/). The Open Database License (ODbL) is a license agreement intended to allow users to freely share, modify, and use this Dataset while maintaining this same freedom for others, provided that the original source and author(s) are credited. Brecht Verstraete, Olivier Lachenaud, Petra De Block, Arne Mertens, Steven B. Janssens, Judith R. Schepers, Steven Jansen, Elmar Robbrecht

Supplementary material 2 Specimens used in the morphological analysis, with taxon names, voucher information, specimen links, and investigated plant parts Data type csv https://binary.pensoft.net/file/1570140This dataset is made available under the Open Database License (http://opendatacommons.org/licenses/odbl/1.0/). The Open Database License (ODbL) is a license agreement intended to allow users to freely share, modify, and use this Dataset while maintaining this same freedom for others, provided that the original source and author(s) are credited. Brecht Verstraete, Olivier Lachenaud, Petra De Block, Arne Mertens, Steven B. Janssens, Judith R. Schepers, Steven Jansen, Elmar Robbrecht

Supplementary material 3 Dataset used in the biogeographical and climate analyses, containing the coordinates of specimens of *Rhodopentas bussei*, *Rhodopentas parvifolia*, *Sacosperma paniculatum*, and *Sacosperma parviflorum* Data type csv https://binary.pensoft.net/file/1570141This dataset is made available under the Open Database License (http://opendatacommons.org/licenses/odbl/1.0/). The Open Database License (ODbL) is a license agreement intended to allow users to freely share, modify, and use this Dataset while maintaining this same freedom for others, provided that the original source and author(s) are credited. Brecht Verstraete, Olivier Lachenaud, Petra De Block, Arne Mertens, Steven B. Janssens, Judith R. Schepers, Steven Jansen, Elmar Robbrecht

Supplementary material 4 Bayesian phylogenetic tree of Knoxieae based on ITS, *rps16*, and *trnL-F* Data type pdf Explanation note Posterior probabilities are given at the nodes. *Sacosperma paniculatum* is indicated in bold. https://binary.pensoft.net/file/1570142This dataset is made available under the Open Database License (http://opendatacommons.org/licenses/odbl/1.0/). The Open Database License (ODbL) is a license agreement intended to allow users to freely share, modify, and use this Dataset while maintaining this same freedom for others, provided that the original source and author(s) are credited. Brecht Verstraete, Olivier Lachenaud, Petra De Block, Arne Mertens, Steven B. Janssens, Judith R. Schepers, Steven Jansen, Elmar Robbrecht

Supplementary material 5 Maximum likelihood tree of Knoxieae based on ITS, *rps16*, and *trnL-F* Data type pdf Explanation note Bootstrap values are given at the nodes. *Sacosperma paniculatum* is indicated in bold. https://binary.pensoft.net/file/1570143This dataset is made available under the Open Database License (http://opendatacommons.org/licenses/odbl/1.0/). The Open Database License (ODbL) is a license agreement intended to allow users to freely share, modify, and use this Dataset while maintaining this same freedom for others, provided that the original source and author(s) are credited. Brecht Verstraete, Olivier Lachenaud, Petra De Block, Arne Mertens, Steven B. Janssens, Judith R. Schepers, Steven Jansen, Elmar Robbrecht
