# Supplementary figures and images for: Molecular and morphological evidence supports transferring Sacosperma (Rubiaceae, Rubioideae) from Spermacoceae to Knoxieae
Source: PhytoKeys. 2026 Mar 26;272:79–106. doi: 10.3897/phytokeys.272.179527 (PMC13047382; doi:10.3897/phytokeys.272.179527)

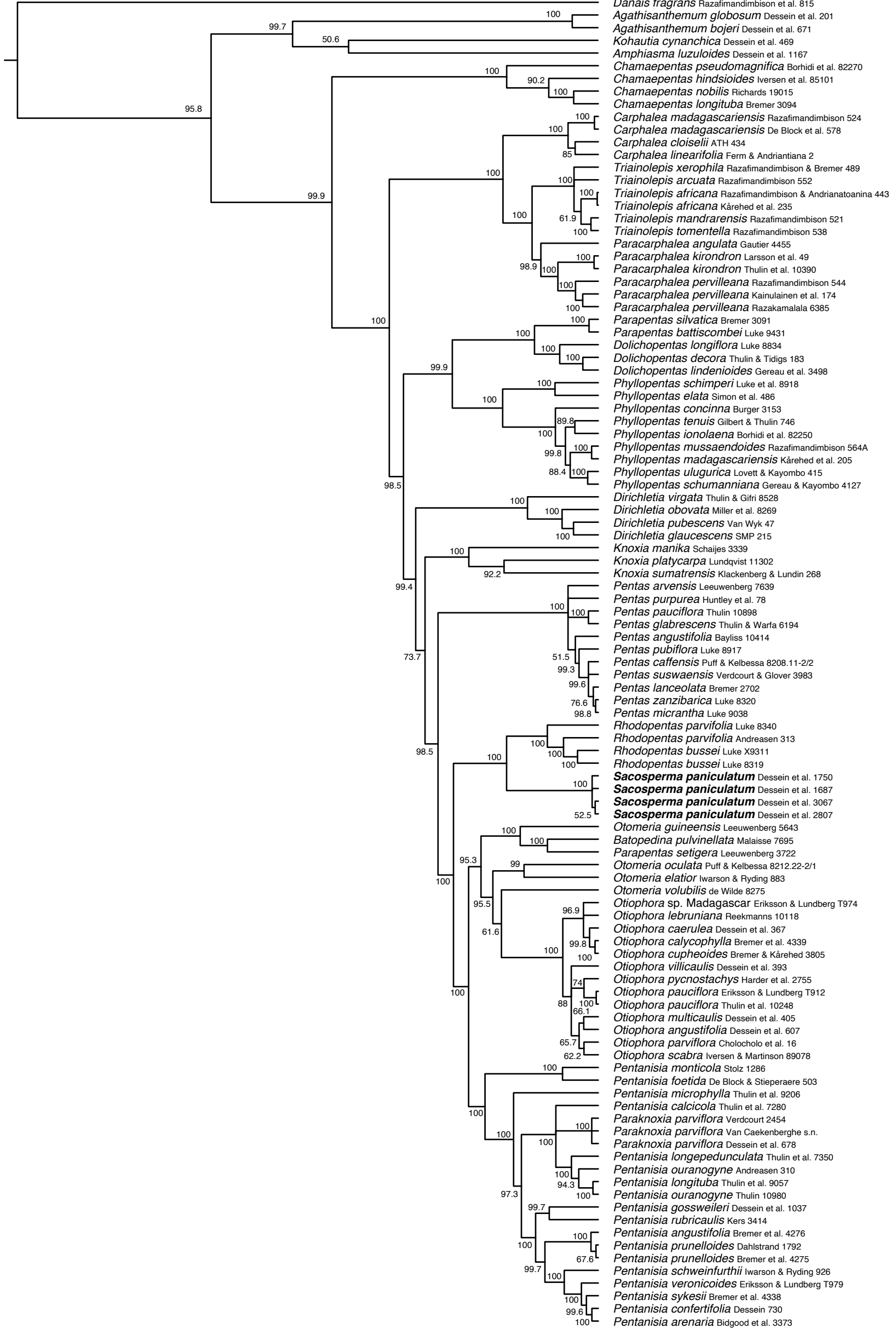

Supplement: Supplementary material 4 — Bayesian phylogenetic tree of Knoxieae based on ITS, rps16, and trnL-F [file phytokeys-272-079_article-179527__-s004.pdf]

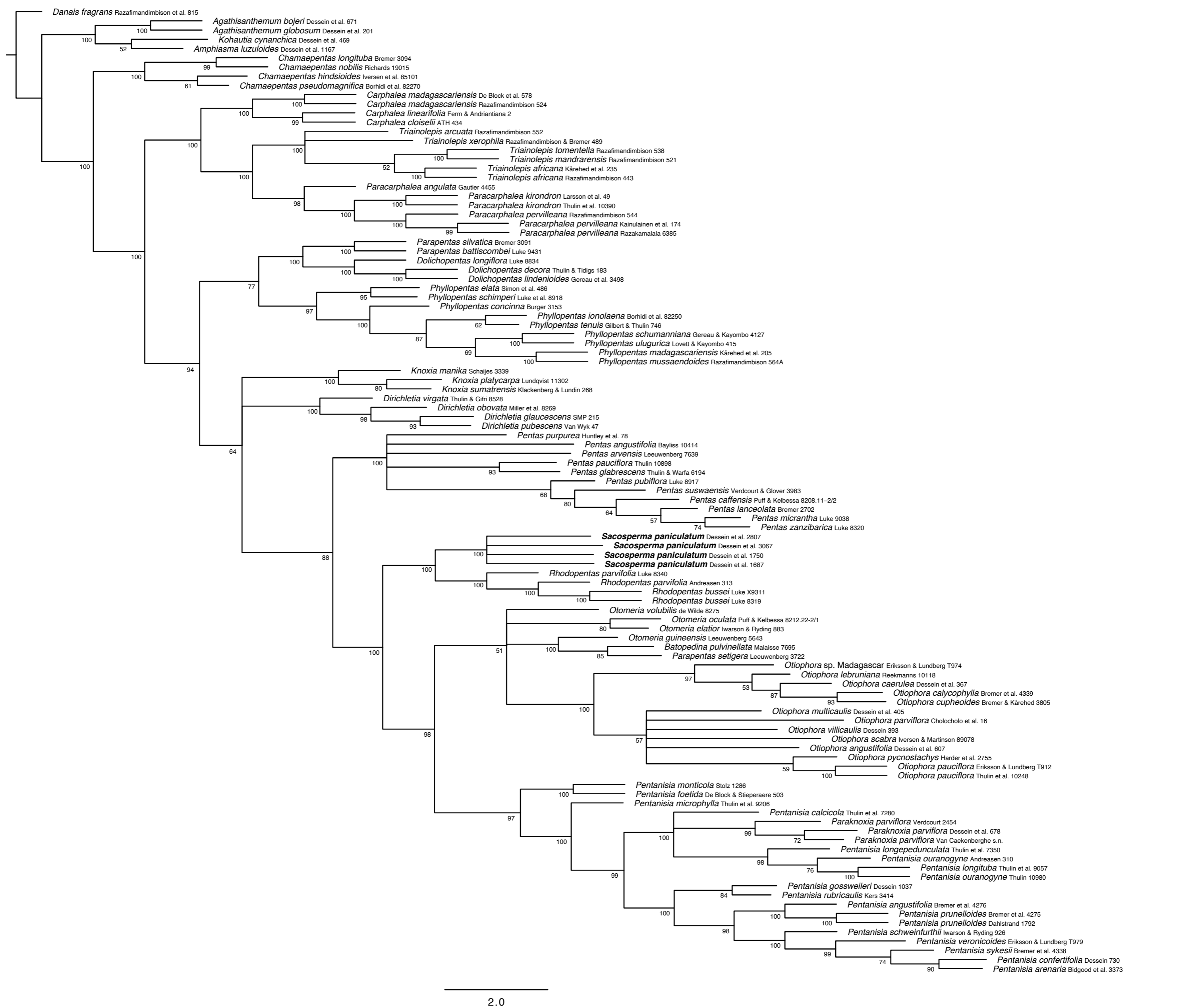

2.0

Supplement: Supplementary material 5 — Maximum likelihood tree of Knoxieae based on ITS, rps16, and trnL-F [file phytokeys-272-079_article-179527__-s005.pdf]
